# Analysis of dual energy spectral CT and pathological grading of clear cell renal cell carcinoma (ccRCC)

**DOI:** 10.1371/journal.pone.0195699

**Published:** 2018-05-01

**Authors:** Jinyan Wei, Jianhong Zhao, Xueling Zhang, Dan Wang, Wenjuan Zhang, Zhiping Wang, Junlin Zhou

**Affiliations:** 1 Department of Radiology, Lanzhou University Second Hospital, Lanzhou, PR China; 2 Institute of Urology, Lanzhou University Second Hospital, Key Laboratory of Gansu Province for Urological Diseases, Clinical Center of Gansu Province for Nephrourology, Lanzhou, PR China; Northwestern University Feinberg School of Medicine, UNITED STATES

## Abstract

**Objective:**

To discuss the dual energy spectral computer tomography (CT) imaging features of the pathological grading of clear cell renal cell carcinoma (ccRCC) and the correlation between spectral CT imaging features and pathology.

**Materials and methods:**

We performed analyses of 62 patients with confirmed diagnosis of ccRCC. All patients underwent non-enhanced CT and dual-phase (cortex phase, CP and parenchyma phase, PP) contrast-enhanced CT with dual energy spectral mode. The subjects were pathologically divided into two groups: low-grade group (Fuhrman 1/2) and high-grade group (Fuhrman 3/4). The CT value of each lesion was measured on the monochromatic image at 70 keV. The normalized iodine concentrations (NIC) and the slope of the spectrum curve were calculated. The qualitative morphological parameters, including tumor shape, calcification, pseudocapsule, necrosis, and enhancement mode, were compared between the two groups. The quantitative data were compared using Student’s t-test, and the enumeration data were analyzed using the chi-square test between low-grade and high-grade ccRCC.Receiver operating characteristic curve was used to determine the sensitivity and specificity of the quantitative parameters in two groups.

**Results:**

The CT value, NIC, and mean slope of the low-grade group were increased compared with the high-grade group during CP (*P =* 0.001, *P* = 0.043, and *P<*0.001, respectively). The CT did not differ significantly during PP (*P =* 0.134); however, the NIC and mean slope varied considerably in the low grade compared with the high-grade group (*P* = 0.048, *P* = 0.017, respectively). The CT threshold value, NIC, and slope had high sensitivity and specificity in differentiating low-grade ccRCC from high-grade ccRCC. The tumor shape, pseudocapsule, and necrosis differed significantly between the two groups (*P<*0.05).

**Conclusions:**

Dual energy spectral CT with the quantitative analysis of iodine concentration and qualitative analysis of morphological characteristics increases the accuracy of diagnosing the pathological grade of ccRCC.

## Introduction

Renal cell carcinoma (RCC) is the most common primary malignant tumor of the kidney and accounts for approximately 85–90% of malignant kidney tumors. Clear cell renal cell carcinoma (ccRCC) is the most common subtype of RCC, accounting for approximately 70% of cases[1].Given the high morbidity and mortality of ccRCC[2], an earlier diagnosis and treatment is essential for improving patient survival and quality of life. Numerous factors affect the prognosis of ccRCC, and Fuhrman nuclear grade is an independent index for assessing prognosis[3]. ccRCC is divided into 1–4 grades according to Fuhrman nuclear grade. To increase the reproducibility and reduce the intra-/inter-observer variability, 4-tiered Fuhrman grading system can be merged into simplified 2-tiered system: grade 1and 2 as low grade (well-differentiated tumors), and grade 3 and 4 as high grade (poorly differentiated tumors), which performed similarly to the prognostic ability of the traditional[4]. Fuhrman grade 1 is the least aggressive type, with grade 4 being the most aggressive[5]. Resection is currently an effective treatment for RCC, including radical and partial nephrectomy. To evaluate the malignant degree of RCC and to select the operation scheme, preoperative pathological classification of RCC is of vital importance.

Clinically, multislice CT is the most common radiological technique for diagnosing kidney cancer. CT performance of tumor characteristics contributes to increasing the detection rate and diagnosis accuracy, aids in clinical decision-making and serves as the primary basis for staging and treatment response assessment. The diagnosis of ccRCC can usually be based on typical CT imaging characteristics, and these characteristics maintain the high diagnostic accuracy of ccRCC[6]. Nevertheless, the image-based distinction grading of ccRCC is often controversial, because of findings that are not shown to be pathognomonic with dynamic contrast-enhanced multidetector row CT[7–9]. To access conventional hybrid energy images, dual energy spectral CT can obtain single energy images within the range of 40–140 keV. Furthermore, the material separation images achieve the transformation of the diagnosis pattern from single to multiple parameters and serves as a hybrid for single energy imaging[10, 11]. Burgeoning evidence indicates that dual-energy spectral CT with iodine quantification enables a reliable distinction renal lesions, with higher accuracy compared with conventional enhancement measurements[12, 13].

The present study aims to investigate the dual energy spectral CT imaging features of the pathological grading of ccRCC and the correlation between spectral CT imaging features and pathology.

## Materials and methods

### Patients

The Ethics Committee at Lanzhou University Second Hospital approved this retrospective study, and all patients provided written informed consent. From May 2014 to December 2015, 180 patients known or suspected for renal tumors underwent non-enhanced CT and dual-phase contrast-enhanced CT with the dual energy spectral mode. One hundred and eighteen patients were excluded from the study because they either did not have ccRCC or their histological finding provided inadequate confirmation. A final cohort of 62 patients [44 males, 18 females; median age 55±12 (24–76 years)] was included in the present study. 29 patients suffered from lower back pain and discomfort while 18 patients showed painless gross hematuria. In addition, 7 patients accompanied frequent urination, urgent urination and odynuria. 14 patients had no obvious discomfort and the renal lesion detected in health examination.

All sixty-two patients were confirmed to have ccRCC by surgical and pathological assessment. The subjects were divided into two groups based on the histological characteristics of hematoxylin-eosin (HE) staining: low-grade group (Fuhrman 1/2; 35 patients) and high-grade group (Fuhrman 3/4; 27 patients).

### CT examination

Triple-phase CT (i.e., unenhanced and two-phase contrast-enhanced CT examinations) was performed using a Discovery CT750 HD CT system (GE Healthcare, Waukesha, WI, USA). The scan range included the superior border of the liver up to the anterior superior spine. Unenhanced images were acquired using the conventional helical scan mode at 120 kVp tube voltage. The patients were then injected with nonionic contrast medium (iohexol, 300 mg iodine/mL) via antecubital venous access at a rate of 3.5–4.0 mL/s for a total of 80–100 mL (1.2 mL/kg of body weight) during the cortex phase (CP) and parenchyma phase (PP). CP scanning began 20 s after the trigger attenuation threshold (50 HU) achieved the level of the supra-celiac abdominal aorta. PP scanning began at a delay of 60 s after CP scanning. CP and PP scanning were performed in the spectral imaging mode with fast tube voltage switching between 80 kVp and 140 kVp on adjacent views during a single rotation. Other scanning parameters were as follows: 0.625 mm collimation thickness, 600 mA tube current, 0.6 s rotation speed, 0.983 helical pitch, 1.25 mm reconstruction thickness, and inter-slice spacing. CT images were reconstructed by projection-based material-decomposition software using a standard reconstruction kernel.

### Quantitative analysis

All measurements were performed on an advanced workstation (AW4.6,Discovery CT 750 HD,GE Healthcare) with a Gemstone Spectral Imaging (GSI) viewer. Circular or elliptical regions of interests (ROI) with an area of approximately 100 mm^2^ were marked on the lesions and aorta with a default of 70 keV for monochromatic images. The ROIs encompassed as much of the enhancing areas of the lesions as possible. Areas containing necrosis, calcification, and large vessels were carefully avoided. To ensure consistency, all measurements were performed thrice at different image levels, and average values calculated. For all measurements, the size, shape and position of the ROIs were maintained consistently between the two phases. The GSI Viewer software automatically calculated the CT attenuation values and iodine (water) and water (iodine) concentrations for the lesions and aorta. Two recently introduced parameters were derived from the iodine concentration measurements and monochromatic images: (a) the normalized iodine concentration (NIC) was calculated as NIC = IC_lesion_/IC_aorta_, where IC_lesion_ and IC_aorta_ are the iodine levels in the lesions and the aorta; iodine concentrations in the lesions were normalized to those of the aorta to minimize variations in patients; (b) the slope of spectrum curve was calculated as slope = (CT_40keV_-CT_70keV_)/30, where CT_40keV_ and CT_70keV_ are the CT attenuation values of the tumors on 40 keV and 70 keV monochromatic images, respectively. Finally, the tumor size was measured as the maximum diameter on the transverse slice.

### Qualitative analysis

**Fig 1:**
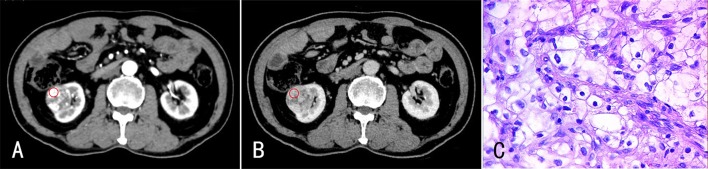
A 68-year-old male with ccRCC (Fuhrman 1) in the right kidney. The enhanced CT images revealed a small circular mass in the right kidney. The lesion exhibited significant enhancement during CP (A), and the enhancement degree was reduced during PP (B). (C) HE staining (×400). The tumor cells were round with small or no nucleoli.

**Fig 2:**
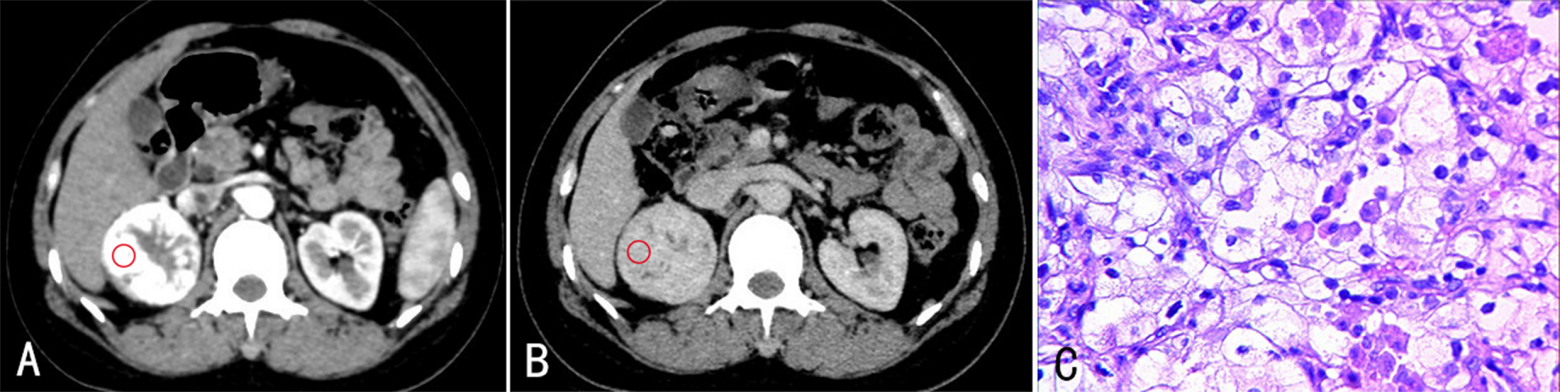
A 58-year-old female with ccRCC (Fuhrman 2) in the right kidney. The enhanced CT images revealed a round mass in the right kidney. The lesion had significant and heterogeneous enhancement during CP (A), and the enhancement degree was reduced during PP (B). (C) Stained with HE (×400). The tumor cells were round in shape. Some small nuclei were increscent, and some were not evident.

**Fig 3:**
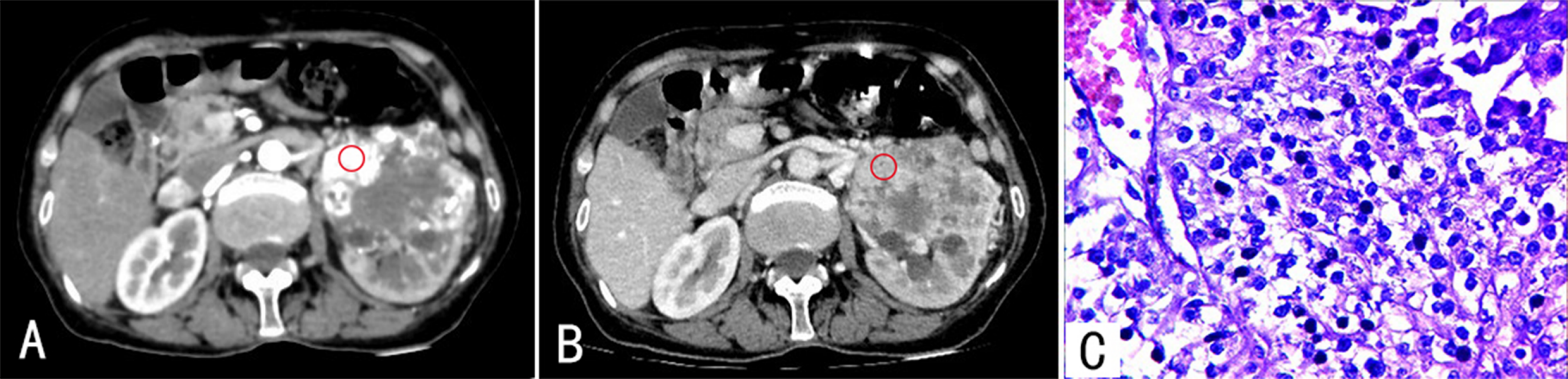
A 74-year-old male with ccRCC (Fuhrman 3–4) in the left kidney. The enhanced CT images revealed a large mass with an irregular shape in the left kidney. The lesion had heterogeneous enhancement during CP (A) and PP (B), and the necrotic area exhibited no enhancement. (C) Stained with HE (×400). The tumor cells had an irregular shape. Nuclei were distinctly abnormal, and some had large nucleoli.

Two radiologists with greater than 10 years of experience in abdominal CT diagnosis were blinded to the diagnosis of the lesion, patient information, and the correlative imaging examination results. The following lesion features were reviewed: tumor shape, calcification, psuedocapsule, necrosis, and the enhancement pattern on 70 keV monochromatic images. A tumor with a circular or elliptical shape was considered regular, whereas all other tumors were considered as irregular. Psuedocapsule was noted when low-density ring around the tumor was present. Necrosis was defined as either the presence or absence of areas within the tumor that did not demonstrate contrast enhancement during the cortex and parenchyma phases. The enhancement pattern was described as homogeneous or heterogeneous during CP and PP. Finally, the reviewers characterized each lesion as ccRCC based on the consensus of the imaging findings. Differences among the observers were resolved by means of a consensus conference. The judgment standard of some imaging features was presented in the following images (**Figs 1–3**).

### Statistical analysis

All analyses were performed using SPSS19.0 (Chicago, IL, USA). Quantitative values were recorded as the mean±standard deviation. The quantitative data were compared using Student’s t-test, and the enumeration data were analyzed using the chi-square test between low-grade and high-grade ccRCC. *P*<0.05 indicates significance.

Receiver-operating characteristics (ROC) curves were generated for the values with statistically significant differences to evaluate their diagnostic efficiency and calculate the cut-off value, sensitivity, and specificity in the maximal Youden's index (YI). Sensitivity was defined as the number of correct diagnoses of low-grade ccRCC divided by the number of proven low-grade ccRCC multiplied by 100. Specificity was defined as the number of correct diagnoses of high-grade ccRCC divided by the number of proven high-grade ccRCC multiplied by 100. The null hypothesis was that the area under the ROC curve (AUC) was 0.5; the alternative was AUC>0.5.

## Results

### Quantitative analysis

**Fig 4:**
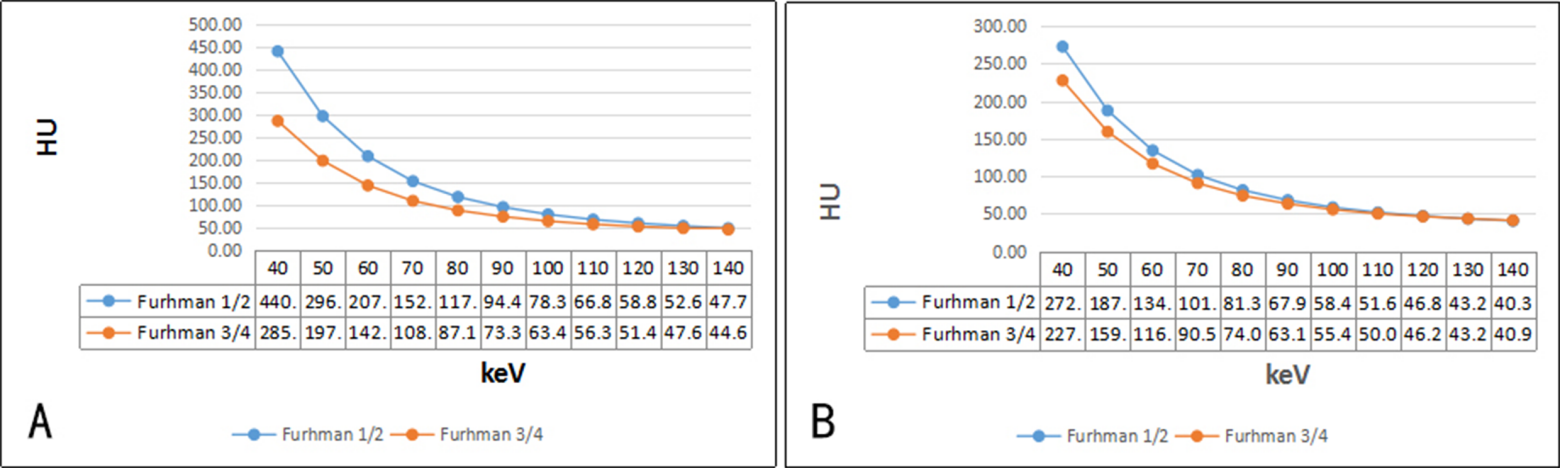
**The fitting spectrum curve for different pathological grades of ccRCC exhibited significant differences during CP (A) and PP (B).** (The slope of spectrum curve was calculated as slope = (CT_40keV_-CT_70keV_)/30, where CT_40keV_ and CT_70keV_ are the CT attenuation values of the tumors on 40 keV and 70 keV monochromatic images).

The mean diameters of well-differentiated and poorly differentiated tumors were 5.08±1.58 and 8.48±2.65 cm, respectively (*P*<0.001). Values for the defined quantitative parameters measured in patients with different pathologic grades of ccRCC are listed in **Table 1**. The CT value of the 70 keV monochromatic image in CP of the low-grade group was higher than the high-grade group, but no significant differences in PP were noted. The low-grade group exhibited a higher NIC ratio and mean slope in CP and PP compared with the high-grade group. The spectrum curves of the two groups during CP and PP are presented in **Fig 4**. The water concentration ratios of the two groups were not statistically different during both phases (*P*>0.05).

**Table 1:** Quantitative assessment of different pathological grading of ccRCC with CT spectral imaging.

Group	CP			PP		
	low grade	high grade	*P*-value	low grade	high grade	*P*-value
70keV (HU)	151.89±37.38	108.40±17.82	0.001	100.34±18.52	90.57±10.85	0.134
NIC	0.47±0.17	0.31±0.11	0.043	0.83±0.22	0.68±0.11	0.048
Slope	5.99±1.71	3.71±0.85	<0.001	3.51±0.83	2.57±0.59	0.017
Water Concentration Ratio	1.02±0.01	1.01±0.01	0.493	1.00±0.01	1.00±0.01	0.781

NIC, normalized iodine concentration, NIC was calculated as NIC = IC_lesion_/IC_aorta_, where IC_lesion_ and IC_aorta_ are the iodine levels in the lesions and the aorta; Slope was calculated as slope = (CT_40keV_-CT_70keV_)/30, where CT_40keV_ and CT_70keV_ are the CT attenuation values of the tumors on 40 keV and 70 keV monochromatic images; CP, cortex phase; PP, parenchyma phase. Data are mean±standard deviation *P<*0.05 indicates a statistically significant difference between low-grade and high-grade ccRCC.

### The diagnostic performance of quantitative parameters

**Fig 5:**
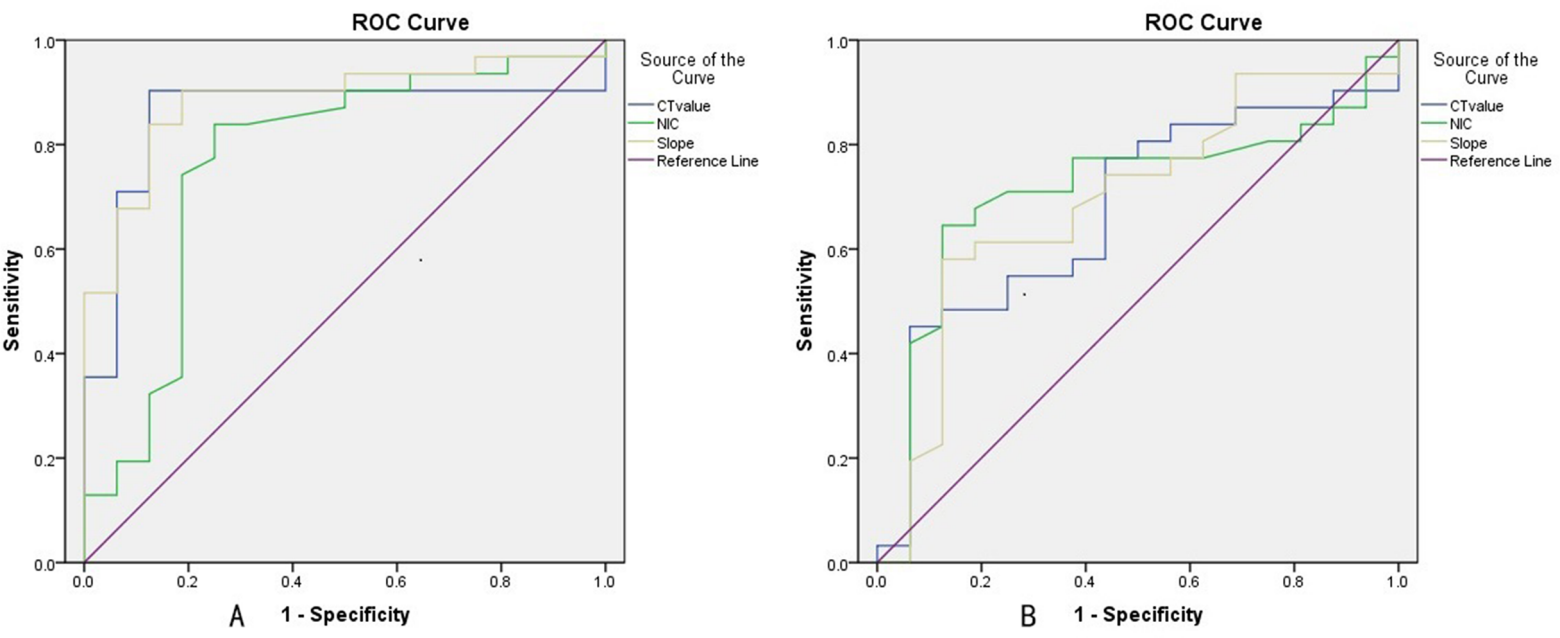
ROC curves for different parameters in differentiating pathological grades of ccRCC during CP (A) and PP (B).

The ROC curves of different parameters for differentiating the pathologic grading of ccRCC are presented in **Fig 5**. The optimal thresholds of quantitative parameters to diagnose the grading of ccRCC are listed in Table 2. The AUC for the spectrum curve slope (0.885) during the CP was greater than that during the PP, and the sensitivity (90.3%) was increased compared with sensitivities for the other quantitative parameters in diagnosing the pathologic grading of ccRCC (**Table 2**).

**Table 2:** The optimal threshold of quantitative parameters to diagnose the grading of ccRCC.

Group	AUC	Threshold	Sensitivity	Specificity
CP				
70keV	0.857	109.24	87.1(30)	87.5 (24)
NIC	0.771	0.35	83.9 (29)	75.0 (20)
Slope	0.885	4.03	90.3 (32)	87.5(24)
PP				
70keV	0.675	107.98	45.2 (16)	93.7 (25)
NIC	0.710	0.85	58.1 (20)	87.5 (24)
slope	0.693	3.65	58.1 (20)	87.5 (24)

NIC, normalized iodine concentration; CP, cortex phase; PP, parenchyma phase. Threshold 70keV are cited in Hounsfield (HU). Threshold NIC in CP and PP are represented as mg/mL. Sensitivity values are demonstrated as %. Data in parentheses are numbers of low-grade ccRCC lesions (n = 35) used to calculate percentages. Specificity values are cited as %. Data in parentheses are numbers of low-grade ccRCC lesions (n = 27) used to calculate percentages.

### Qualitative analysis of monochromatic images

The results of the qualitative analysis of monochromatic images are provided in **Table 3**. The shape,　pseudocapsule, and area of necrosis differed between low-grade ccRCC and high-grade ccRCC (*P*<0.05). The calcification and enhancement mode between the two groups were similar (*P*>0.05). Binary logistic regression showed only necrosis was related to pathological grading of ccRCC (*P*<0.05).

**Table 3:** Qualitative assessment with monochromatic image of different pathological ccRCC during CP and PP.

CT signs		A (35)		B (27)		*χ*^2^-value	*P*-value
		n	%	n	%		
Shape	regular	27	77.1	8	29.6	16.362	<0.001
	no	8	22.9	19	70.4		
Calcification	yes	11	31.4	4	14.8	3.981	0.137
	no	24	68.6	23	85.2		
Pseudocapsule	yes	26	74.3	7	25.9	18.258	<0.001
	no	9	25.7	20	74.1		
Necrosis	yes	17	48.6	24	88.9	11.467	0.003
	no	18	51.4	3	11.1		
Enhancement pattern	Homogeneous	10	28.6	3	11.1	3.004	0.223
	no	25	71.4	24	88.9		

Data are numbers of lesions and percentages. *P<*0.05 indicates a statistically significant difference between low-grade ccRCC and high-grade ccRCC.

In the qualitative analysis of the imaging features observed during the combined CP and PP, a sensitivity and specificity of 80% (28 of 35 low-grade ccRCC) and 77.8% (21 of 27 high-grade ccRCC), respectively, were achieved for differentiating between low- and high-grade ccRCC. In conclusion, the quantitative parameters analysis with CT spectral imaging compared with qualitative CT image analysis improved the sensitivity from 80% to 90.3% and the specificity from 77.8% to 87.5%.

## Discussion

RCC is classified into 11 subtypes by the WHO [14] based on morphological characteristics of the tumor tissue combined with genetic alterations and tumor origin, and ccRCC is the most common subtype. The diagnosis of ccRCC is generally based on typical CT imaging characteristics. Tumor necrosis is common, and the density is heterogeneous. Contrast-enhanced CT demonstrates that the lesions have significant and heterogeneous enhancement during CP, and the enhancement degree is reduced during PP[6]. Given the high incidence of ccRCC, combining CT features with clinical manifestations can provide an accurate preoperative diagnosis about most ccRCC; however, conventional CT has substantial limitations in evaluating the pathological grading of ccRCC.

Our study demonstrated that the CT value of the 70 keV monochromatic image of the low-grade tumor was increased compared with the high-grade tumor during CP, but no significant differences were noted during PP. NICs varied significantly between the two groups during CP and PP. According to previous studies, tumor blood supply types correlated with the pathological grade[15–17]. A well-differentiated ccRCC had relatively mature arteries, rapid blood flow, and enhanced vascular permeability. Thus, the contrast agents readily entered into the tumor tissue. Poorly differentiated ccRCC exhibits a high degree of malignancy, accelerated tumor growth rate, and incomplete new blood vessel structures with uneven distribution. These lesions exhibit hemodynamic abnormalities and an arteriovenous shunt, which leads to the uneven distribution of the blood supply[18, 19]. Tumor angiogenesis and CT enhancement correlate with microvessel density in RCC[16, 20]. The expression of vascular endothelial growth factor increases as the tumor grade increases; hence, the regeneration of a large number of blood vessels and the microvascular density increases, which optimize conditions for rapid tumor growth[21]. However, the reduced microvessel density of the tumor center and the increased tumor size result in insufficient central blood supply to the tumor, leading to necrosis and cystic change[22]. Therefore, high-grade ccRCC exhibit a reduced blood supply compared with low-grade ccRCC. The result was similar to that of Asayama et al. In that study, the number of unpaired arteries increased as the histological grade progressed from well-differentiated HCC to moderately differentiated HCC but decreased as the tumor grade progressed from moderately differentiated HCC to poorly differentiated HCC[17].

The slope of the spectrum curve exhibited significant differences between low-grade and high-grade ccRCC during CP and PP. The spectral curve reflects the material CT value varying with the energy of X-ray and the absorption characteristics to the different energy of X-ray. Various substances exhibit changes in chemical molecular structures, and different chemical molecules have modified energy attenuation curves[23]. Thus, we can distinguish the chemical composition of substances by comparing the slope of the spectrum curve[24]. Given the blood supply of the poorly differentiated, Fuhrman 3/4 ccRCC lesions, the enhancement degree is reduced. Hence, the spectral curve slope is minimized.

The CT signs of a monochromatic image are differently expressed in different grades of ccRCC[25, 26]. High-grade ccRCC exhibit a larger size compared with low-grade ccRCC. This CT sign predicts a larger tumor size and increased malignancy[27]. Tumor biology diversifies with differentiation degree. The margin of the well-differentiated, Fuhrman1/2 ccRCC lesions was regular, and a psuedocapsule was often observed. The poorly differentiated, Fuhrman3/4 ccRCC lesions were aggressive with irregular margins, the absence of a psuedocapsule, and unclear boundaries. Previous studies revealed histological necrosis as an independent predictor of ccRCC prognosis[28, 29]. Tumor necrosis results when a tumor outgrows its blood supply. Given that poorly differentiated tumors proliferate rapidly and the large tumor size, the microvessel density of the tumor center decreased, resulting in necrosis. High-grade ccRCC is a heterogeneous tumor that exhibits necrosis, fibrosis, hemorrhage and edema[30]. In the enhancement mode of ccRCC, the contrast agent was “early into, early exit”, and no significant differences were noted between high-grade ccRCC and low-grade ccRCC[31]. A previous study[15] demonstrated that the early-stage enhancement rate of the lesion was suggestive of the density of microvessels, indicating that tumors with abundant microvessels tend to exhibit rapid improvement in early phases.

ROC curves analysis in our study revealed that the quantitative parameters exhibited increased sensitivity and specificity in CP compared with PP for distinguishing well-differentiated ccRCC from poorly differentiated ccRCC. Given that ccRCC is a tumor with rich blood supply, the enhanced scan exhibit significant enhancement during CP, and the degree of enhancement is reduced during PP. The best quantitative parameter was the slope of the spectrum curve in CP. The threshold slope of 4.03 yielded a sensitivity and specificity of 90.3% and 87.5%, respectively. Compared with qualitative imaging analysis, quantitative analysis with spectral CT improved both the sensitivity (from 80% to 90.3%) and specificity (from 77.8% to 87.5%). The threshold values evaluated in our study were based on specific populations, and the accuracy of these values require further confirmation using larger sample sizes in future studies.

However, our study had some limitations. First, the study sample size was small, and the results were preliminary, which necessitate verification by additional studies performed with a larger number of lesions. Second, this study focused on discriminating well-differentiated ccRCC from poorly differentiated ccRCC. Future investigations with other cases of different types of renal tumors are needed to draw broader conclusions. Third, the correlation between the histopathological and spectral CT quantitative parameters and imaging features was involved in this study but not considered further.

In summary, dual energy spectral CT with quantitative analysis of iodine concentration and qualitative analysis of morphological characteristics and the correlation between spectral CT imaging features and pathology may help increase the accuracy in differentiating the pathological grading of ccRCC.

## Supporting information

S1 File The raw datas were included in the file. (RAR)
